# Does Human Papillomavirus Play a Causative Role in Prostate Cancer? A Systematic Review Using Bradford Hill’s Criteria

**DOI:** 10.3390/cancers15153897

**Published:** 2023-07-31

**Authors:** Ridwan Opeyemi Bello, Lily Willis-Powell, Olivia James, Avyay Sharma, Elizabeth Marsh, Libby Ellis, Kevin Gaston, Yusra Siddiqui

**Affiliations:** 1 School of Human Sciences, College of Science and Engineering, University of Derby, Derby DE22 1GB, UK; 100613994@unimail.derby.ac.uk (R.O.B.); e.marsh@derby.ac.uk (E.M.); 2 School of Medicine, University of Nottingham, Nottingham NG7 2QL, UKkevin.gaston@nottingham.ac.uk (K.G.)

**Keywords:** prostate cancer, human papillomavirus, HPV

## Abstract

**Simple Summary:**

Prostate cancer is a major cause of cancer-related deaths globally, yet its causes remain unclear. While human papillomavirus (HPV) is known to be associated with other cancers, such as cervical cancer and anal cancer, investigation of its connection to prostate cancer has yielded mixed results. This systematic review aimed to assess the relationship between HPV and prostate cancer using Bradford Hill’s criteria. Out of 482 studies screened from PubMed, 60 were included and evaluated. Although the strength of association was not strong and certain criteria were not met, the review identified plausible biological mechanisms and reported the presence of HPV in prostate cancer tissues. However, the overall quality of evidence remains low, and further high-quality studies are needed to establish a definitive link. These findings have implications for understanding prostate cancer and guiding future research efforts.

**Abstract:**

Globally, prostate cancer is the fifth most common cause of cancer-related death among men, and metastatic castration-resistant prostate cancer has a high cancer-related mortality rate. However, the aetiology of this disease is not yet fully understood. While human papillomavirus (HPV) has been associated with several types of cancer, including cervical, anal, and oropharyngeal cancers, studies investigating the relationship between HPV and prostate cancer have shown mixed results. This systematic review aimed to evaluate the causative association between HPV and prostate cancer using Bradford Hill’s criteria. A comprehensive search of PubMed was conducted, and 60 out of 482 studies were included in the review. The included studies were evaluated based on nine Bradford Hill criteria, and information on the identification and transmission of the virus and potential oncogenic mechanisms was also extracted. The strength of association criterion was not met, and other criteria, such as consistency and coherence, were not fulfilled. However, biological plausibility was supported, and potential oncogenic mechanisms were identified. While some studies have reported the presence of HPV in prostate cancer tissues, the overall quality of evidence remains low, and the association between HPV and prostate cancer is weak. Nevertheless, the prostate is a potential reservoir for the transmission of HPV, and the HPV E6 and E7 oncoproteins and inflammation are likely to be involved in any oncogenic mechanisms. Further studies with a higher level of evidence are needed to establish a definitive link between HPV and prostate cancer.

## 1. Introduction

Prostate cancer (PCa) is the second most common form of cancer among men, with an estimated 1.4 million new cases and 375,000 deaths worldwide in 2020 [1] PCa incidence increases with age and the disease is most commonly diagnosed in men over 50 [2]. In addition, age, race, genetics, and a positive family history of PCa are nonmodifiable risk factors strongly associated with PCa development [3,4,5]. Modifiable risk factors such as metabolic syndrome, smoking, diet, obesity, physical activity, and exposure to ultraviolet rays may impact the risk of developing PCa and PCa mortality [3,5,6,7]. Benign prostatic hyperplasia (BPH) is a noncancerous condition characterised by an enlargement of the prostate gland. While BPH and PCa often occur together, BPH is not recognised as a confirmed risk factor for PCa [8,9]. Instead, BPH may increase the likelihood of detecting incidental cancer, despite not being directly responsible for increasing the risk of developing PCa [8,9]. Additionally, several studies have found the presence of pathogens in PCa tissues, including human papillomaviruses (HPVs), human cytomegalovirus, Epstein–Barr virus, and *Propionibacterium acnes* [10,11,12].

HPV is one of the most common sexually transmitted infections globally, and at least 12 so-called high-risk HPV types have been identified as human carcinogens [13]. HPV 16HPV16 and HPV 18 are the best understood high-risk HPV types. The oncoproteins encoded by high-risk HPV types possess a greater ability to efficiently transform infected cells compared to oncoproteins from other HPV types. The oncoproteins interact with critical cellular targets, disrupting normal cellular processes, and contribute to cell transformation. The E6 and E7 oncoproteins from these viruses are well characterised in terms of their ability to inactivate the p53 and pRB tumour-suppressor proteins, respectively, although they also have a variety of other cellular targets. The HPV E5 protein can also act as an oncoprotein, for example, through the stimulation of Epidermal Growth Factor activity [14,15]. Although it is not part of the normal HPV life cycle, HPV-transformed cancer cells often contain viral DNA integrated into the host genome with retained expression of E6 and E7 [16,17].

The relationship between HPV and PCa has been the subject of much debate and research in recent years, with results from various studies being contradictory. To clarify the connection between these two factors, statistical meta-analysis has been employed [18,19,20]. However, the limitations of this method, including difficulties in evaluating study methodologies, accounting for differences in study populations, and publication bias, have left many questions unanswered. To address these limitations, this systematic review aims to evaluate the causal relationship between HPV and PCa using Bradford Hill criteria postulates [21], a set of well-established criteria for evaluating causality. The review will analyse the available literature on the topic by applying the expanded version of Bradford Hill’s criteria for causality. These criteria, originally consisting of the strength of association, consistency, specificity, temporality, biological gradient, plausibility, coherence, experiment, and analogy, have been expanded to include the latest advances in the study of oncoviruses such as virus identification, transmission mechanisms, and oncogenic processes.

Our investigation is premised on the idea that high-risk HPV types could play a causative role in PCa. As HPV infections can be prevented through vaccination, investigating their potential role in PCa is of critical importance.

## 2. Methodology

### 2.1. Literature Search

A comprehensive examination of available research studies was carried out following the PRISMA guidelines for Preferred Reporting Items in Systematic Reviews and Meta-Analyses [22]. A search was conducted, without any time restriction, using the PubMed database with the specified search terms: (Prostate Cancer OR Prostate cancers OR Prostate Carcinoma OR Prostate Carcinomas OR prostate tumour OR prostate tumours OR prostate adenocarcinoma OR prostate adenocarcinomas OR intraepithelial prostatic neoplasia OR intraepithelial prostatic neoplasias OR benign prostatic hyperplasia [MeSH Terms]) AND (HPV OR humanpapillomavirus OR human papillomavirus OR human papilloma virus OR alpha papillomavirus [MeSH Terms]). We only included studies that produced primary data, so all reviews and meta-analyses were discounted. This search was conducted on 16 May 2023. This review was not register for PRISMA.

### 2.2. Paper Selection

**Figure 1:**
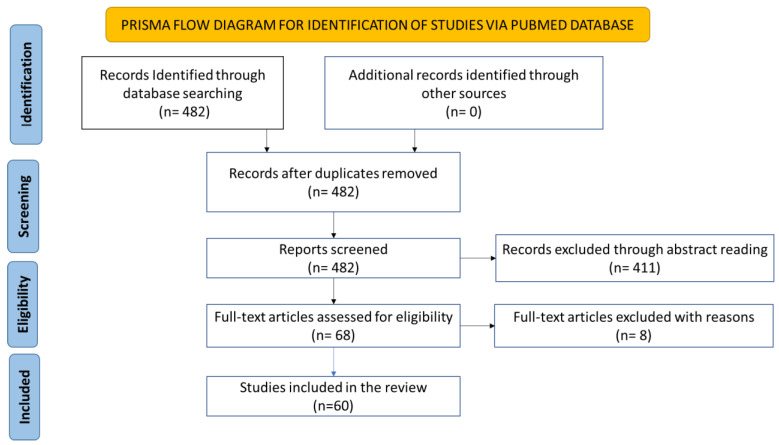
PRISMA flowchart for selecting and screening the resultant papers of the initial literature search.

The initial search resulted in 482 papers. We screened the abstracts of the 482 papers in terms of the inclusion and exclusion criteria and relevance to the study (Table 1). All abstracts were double-checked for relevance. As seen in Figure 1, this method ended with 68 papers deemed relevant and sufficient for this systematic review. Thereafter, a full-text review was performed. At this stage, 8 papers were excluded. Reasons for exclusion were a lack of quality in the study design; a main focus on other infectious pathogens or the prostate microenvironment as a whole and lack of HPV focus; and the paper being in a language other than English.

### 2.3. Quality Criteria Using the Newcastle–Ottawa Quality Assessment Scale

The relevant 60 papers were screened for quality using the Newcastle–Ottawa Quality Assessment Scale [23] (Wells et al., 2021). These criteria include a set of questions that differ depending on whether a case-control study or a cohort study is being assessed. Each study was awarded a maximum of one star for each question that it met. The categories of questions, including Selection, Comparability, and Exposure/Outcome, were assessed to evaluate the risk of bias and to assess the overall strength of the evidence. The exposure category has three questions, with the last being irrelevant to this review as it is aimed at questionnaires or interviews. The quality of case-control studies was rated out of a maximum of seven (Table 2), while the quality of cohort studies was rated out of a maximum of eight (Table 3).

### 2.4. Relevant Data Extraction

To extract data to assess the causal relationship between HPV and PCa incidence, each paper that scored above 3 on the Newcastle–Ottawa scale was selected and scrutinised using the nine (9) central postulates of the Bradford Hill criteria. These nine (9) criteria are Strength, Consistency, Temporality, Biological Gradient, Specificity (now defunct), Biological Plausibility, Coherence, Experiment and Analogy. To aid this review, we added specific criteria for means of transmission, identification of the virus, and oncogenic mechanisms to the Bradford Hill criteria.

## 3. Result and Discussion

A total of 60 original papers were reviewed to investigate the causative role of HPV in PCa. Among the 60 studies, 11 (18%) showed a positive association between HPV presence and the development of PCa. Another 10 studies (17%) were uncertain and suggested further research was necessary, while the remaining 39 studies found no association.

### 3.1. Analogy

The analogy criterion is one of the nine criteria proposed by Bradford Hill to assess the causal relationship between an exposure and an outcome. It involves comparing the relationship between two phenomena to a previously established causal relationship. This criterion assumes that if two phenomena are similar in relevant ways, they may have similar causal relationships [21]. In this way, the analogy criterion provides a framework for exploring potential causal relationships in situations where direct evidence may be limited.

In the case of the relationship between HPV and PCa, the analogy criterion may be useful in exploring potential causal links. HPV is a well-established causal factor in several types of cancer, including cervical cancer, penile cancer, anal cancer, vaginal cancer, vulvar cancer, and cancer of the head and neck; for recent reviews, see [85,86]. Therefore, it is an acceptable hypothesis that it may also cause PCa. However, there are significant differences between the cervix and the prostate in terms of tissue architecture and cell types, hormone biology, and disease biology, as well as some similarities. Cervical cancer originates from squamous cells, whereas PCa has a glandular origin. The role of HPV infection in cervical cancer development is strongly associated with the interaction between HPV and squamous epithelial cells, particularly in the transformation (transition) zone reviewed by [87]. This zone encompasses both glandular cells from the endocervix and squamous cells from the ectocervix. Similarly, the prostate exhibits a transition from glandular epithelium to squamous epithelium in the prostatic urethra although only around a quarter of PCa cases are thought to originate from this zone [88,89]. Furthermore, hormonal changes can induce squamous metaplasia in the prostate, where glandular cells undergo a transformation into squamous cells under the influence of hormones [25]. It is important to note that the presence of squamous epithelium and this metaplastic change does not directly imply susceptibility to HPV infection, but it may impact the prostate’s susceptibility to HPV infection and transformation by HPV. Interestingly, within PCa tumours, there are small regions that exhibit squamous differentiation, characterised by cancer cells resembling squamous cells [90]. Although these regions are present, it is important to highlight that PCa predominantly retains its glandular characteristics. Therefore, how HPV affects cervical and prostate cells may not be directly comparable, and the analogy may be weaker.

In addition, while cervical dysplasia and cervical carcinoma are often regarded as different stages of the same progressive disease, PCa and BPH are regarded as two separate diseases, with high-grade Prostatic Intraepithelial Neoplasia (PIN) acknowledged as the main precursor of invasive prostatic adenocarcinoma [91]. In the cervix, high-grade squamous intraepithelial lesions (HSILs) are associated with HPV and possess the potential to advance into cervical cancer if untreated [92]. Endocervical adenocarcinomas, however, have been classified into those related to high-risk HPV and those unrelated to high-risk HPV [93,94]. Cervical adenocarcinomas may therefore be more comparable to prostate adenocarcinoma. Indeed, some PCa studies have found similarities with cervical cancer studies in the association between different types of HPV and the development of benign versus malignant lesions. For example, in one study, high-risk HPV types (16/18) were found in 92% of HPV-positive PCa cases, while LR types (6/11) were found in only 8% [35]. This is similar to cervical cancer, where HPV16 is the most carcinogenic type. In contrast, LR types were found in 64% of HPV-positive BPH controls [35], indicating that there may be a differential effect of different types of HPV on benign versus malignant tissue.

The analogy criterion can also be useful In exploring potential differences in the nature of the HPV DNA in malignant and pre-malignant lesions. The viral genome often persists in genital neoplasms in an integrated state, and this is a key event in the development of cervical cancer. Integration is not detected in benign lesions and clinically healthy tissues that test positive for HPV [30]. Similarly, it has been suggested that PIN and prostatic carcinoma may have a causal relationship with HPV infection, based on the finding of HPV 16HPV16 in 3 out of 23 carcinoma samples, as well as the similarities observed between PIN and cervical intraepithelial neoplasia (CIN) and penile intraepithelial neoplasia [35,79].

There are limitations to the use of the analogy criterion in exploring potential causal relationships. For example, it is important to ensure that the similarities between different diseases are not superficial and that the mechanisms by which exposure may cause an outcome are comparable. It is also important to consider the possibility of confounding factors and other potential explanations for the observed association. The similarities between PCa and cervical cancer in the way that different types of HPVs act suggest that further investigation into the potential role of HPV in PCa is warranted.

### 3.2. Biological Plausibility and Coherence

Plausibility refers to the idea that there must be a theoretical basis to support a potential association between a virus and a disease [21]. This review indicated that it is plausible for HPV to have an oncogenic role in the development of PCa, based on the idea of plausibility in epidemiology and biology. This is supported by evidence of the oncogenic role of HPV in other types of cancer and by many studies that have evaluated the oncogenic properties of the HPV oncoproteins [95,96,97]. The plausibility of this association is further supported by examining the hypothesised oncogenic mechanisms and means of transmission. Therefore, it is plausible that HPV could be a causative agent in PCa.

Coherence is similar in that the cause-and-effect story should make sense with all knowledge available to the researcher [98]. However, incoherence can be found when the literature is conflicting, and many of the studies around HPV and PCa are contradictory. Therefore, coherence is a criterion that is not met for this topic, based on the studies included in this review.

### 3.3. Identification of Human Papillomaviruses in Prostate Tissues

Establishing the identity of an exposure is crucial in determining a causal relationship between an exposure and outcome. Different methods are used to identify HPV in different studies, each with its strengths and limitations. Polymerase chain reaction (PCR) is a widely used method for detecting the HPV genome, wherein specific primers are used to amplify and detect a DNA fragment of interest. In all, 51 studies used PCR as a detection method; a summary and the primer sets are presented in Table 4. However, the choice of primers varies between studies, and this variability can affect the detection rate of HPV. For instance, one study found that primers targeting a 126 bp fragment of the E6 gene of HPV16 had a higher detection rate than primers targeting a 99 bp fragment of the E6 gene, or a consensus primer pair targeting a 450 bp fragment of the L1 gene [53]. The lower detection rate of the L1 gene could be due to it not always integrating into the host cell genome, and longer DNA segments like L1 can be damaged during formalin-fixation and paraffin-embedding, which are common procedures for tissue storage. Indeed, formalin-fixed paraffin-embedded DNA may not always provide results if the PCR product is over 200 bp [99]. Fresh frozen samples are therefore preferred over formalin-fixed samples as they tend to give more consistent results. Furthermore, different primers may have varying degrees of success in detecting the HPV genome. One study found that while 23/29 cases were positive when testing for the L1 region, additional testing with E6/E7 primers revealed another 6 HPV-positive cases [39]. Despite its sensitivity, a limitation of PCR as a method for HPV detection is that it only shows current exposure. Therefore, previously cleared HPV infection and its possible role in cancer development cannot be assessed. Furthermore, not all PCR primers can identify HPV in PCa. It is possible that the negative HPV identification results in some studies could be due to the use of PCR primers that are not sensitive enough to detect HPV in PCa samples. It is also important to note that the viral load of HPV in PCa is generally lower than that in cervical cancer [100], making it more challenging to detect HPV in PCa samples using PCR. To confirm the findings of PCR, gel electrophoresis or hybridisation techniques such as in situ hybridisation (ISH) or Southern blots are often used, although these require a significant amount of viral DNA and can be time-consuming [46]. Furthermore, Sanger sequencing has been utilised to validate the PCR findings [78,79].

Alternative methods of identifying HPV in PCa have been explored because of the limitations of PCR and Southern blotting. In situ PCR has been used to detect high-risk HPVs in the nuclei of PCa cells and is less susceptible to contamination compared to standard PCR [78]. Hybridisation methods have also been used to identify HPV16/18 in PCa [36,55], Additionally, using next-generation gene sequencing, high-risk HPV types 16 and 18 were identified in 12 of 502 invasive PCa cases from the Cancer Genome Atlas [83]. These findings suggest that alternative methods are valuable in identifying HPV in PCa cases.

Serology is another method used to identify HPV infection, by detecting antibodies to HPV capsid proteins in blood samples. One advantage of serology is that it can detect any prior exposure to HPV since antibodies can persist for a lifetime, even after the clearance of HPV DNA [81]. However, serology lacks anatomical specificity, and a positive serology result only suggests a past HPV infection from any part of the body, not exclusively from the prostate [27].

To supplement the information obtained from HPV detection, immunohistochemistry (IHC) can be used to detect specific proteins in the samples, such as the E6/E7 oncoproteins and tumour markers. This additional method can provide a wider picture of the oncogenic processes and of how HPV may interact with other cellular components. For example, oncogenic HPV E7 proteins were identified through IHC in 112 (75%) of 150 PCa cases [72]. Moreover, another study reported that HPV infection was present in 10% (3/30) of cases with adenocarcinoma and 1.1% (1/90) of cases with BPH using IHC [71]. However, this study’s limitations included the absence of a specific antibody for IHC to detect various HPV subtypes and imprecise data on the exact HPV prevalence in the cancer patients versus normal controls [71].

Understanding the possible reasons for discrepancies in results between studies is crucial for accurately assessing the role of HPV in PCa development. This review highlights the importance of considering differences in laboratory techniques when interpreting the results of HPV detection in PCa studies.

## 4. Strength of Association

Strength of association typically refers to the degree to which a larger association indicates a more likely causal relationship. In this review, the strength of association is evaluated based on whether a statistically significant result was found. However, not all studies performed statistical tests, so this information is not available for all of them.

### 4.1. Serology

All accessible published studies based on serology were included, indicating no selection bias in this review. In this systematic review 13 studies used serology as a detection method (Table 5). Only three studies found statistically significant results in seropositivity rates for high-risk HPV types and an increased risk of PCa [27,30,81]. In contrast, opposing results reported that HPV18 was more prevalent in controls rather than PCa cases [60]. Just 3 of the 13 serology studies included performed PCR on PCa tissues. Interestingly, in two of these cases, the PCR results did not support the serology findings [25,28]. However, one of these studies showed consistent results with negative PCR findings [59]. To identify HPV antibodies in serum samples, Zhao et al. used a cutting-edge technique termed seroscreening by microarray [27]. Serum samples from males with PCa were tested, and HPV16 antibodies were found in 48 of 75 samples (64%), compared to 14 of 80 controls (17.5%) (*p* = 0.001).

Sero-epidemiological studies do not provide a clear case for HPV as a cause of PCa. In contrast, HPV-related cervical cancer shows a significant increase in the prevalence of HPV serum antibodies among patients compared to healthy individuals [101].

### 4.2. Polymerase Chain Reaction

All accessible published studies based on PCR were included, indicating no selection bias in this review. Of these, only 22 concluded whether their results were significant or not regarding HPV prevalence in malignant versus benign tissue. Among these, 7 studies reported significant associations between HPV and PCa, with *p* values < 0.05 [24,35,41,43,56,71,83]. The odds ratios (ORs) reported by the studies that used PCR and concluded significant results varied greatly, ranging from 2.3 to 9.88 [24,43,71], indicating that the strength of association between the HPV and PCa varied greatly between the studies. In total, 15 studies reported no significant results, but not all of them reported ORs or *p* values, with some just stating “no significant findings” or “*p* > 0.05” (Table 6).

From the available evidence, it can be concluded that there is no significant difference in the presence of HPV and the risk of developing PCa. Hence, it appears that the strength of association between HPV and PCa is not strong.

### 4.3. Specificity

The original Bradford Hill criteria state that exposure must only cause one disease to support an epidemiological relationship. This is not the case for many exposure–disease relationships, so this criterion is now defunct and was not considered in this review.

### 4.4. Transmission of Virus

The mode of transmission of a pathogen is a critical factor in developing effective preventive strategies. HPV, a sexually transmitted infection (STI), is often linked to sexual behaviour and to an increased risk of anogenital tumours. Interestingly, sexual behaviours including early age at first intercourse and multiple sexual partners may also increase the risk of PCa [102,103]. A significant portion 216/1272 (17.4%) of the sexually active male population in Britain has detectable HPV DNA in their urine [104]. Both men and women who engage in sexual activity are at an increased risk of contracting high-risk HPV types. Sexual transmission of HPV occurs through cell-to-cell contact during sexual activity, but recent evidence suggests that HPV can also spread throughout the body via circulating extracellular vesicles [105,106]. Exosomes and extracellular vesicles have also been implicated in HPV transmission and carcinogenesis [105].

Interestingly, Saudi Arabia’s population has low incidence rates of PCa, which may be associated with genetic and environmental factors, including circumcision, religionreligious practices, and dietary habits [82]. This may support the role of HPV and sexual activity in PCa promotion, as HPV rates are also lower in this population, and there is lower STI prevalence [82,107]. However, PCa incidence is higher in Iran despite similar cultural and religious practices, although the prevalence of HPV in Iranian populations is lower than in the Western world and Africa [108]. Six studies in this review are from Iran, and five of them do not support the association between HPV and PCa with a *p*-value > 0.05 [26,29,31,34,58]. A serological study with PCa samples from the US found no association between HPV16 or 18 infection status and sexual history [48]. The demographic information collected in the study by Rosenblatt et al. [48] was self-reported, which may affect the validity of the results.

Overall, while a possible link between HPV and PCa through sexual activity is suggested, the evidence is not conclusive, and there may be other factors at play.

### 4.5. Temporality

Temporality is an essential causal criterion and states that exposure must precede the onset of disease for a relationship to be causal. Many of the studies in this review are case-control-based, and in these instances, the temporality criterion is not met. Because of the design of a case-control study, it is not possible to look back in time before the cancer was diagnosed and therefore not possible to prove that HPV infection preceded cancer onset. Only six studies in this review meet the temporality criteria and they report conflicting evidence for a link between HPV and PCa; they are a mix of serology and PCR-based studies.

The first study to meet this criterion is a nested case-control study using a Finnish serum bank. The study reported the relative risk of PCa when seropositive for HPV18 as 2.59 (CI 95% = 1.17–5.75) and found the association between HPV18 and PCa to be highly significant (*p* < 0.05) [81]. The risk also tended to be higher for samples taken more than 10 years before diagnosis. However, a similar nested case-control study, using Nordic biobanks and sera of 200,000 men, did not find an association between serologic markers of HPV16, 18, and 33 infections and risk of PCa [38]. In fact, there was a tendency for an inverse association between PCa risk and HPV18 antibody levels (OR 0.49, CI 0.22–1.09). Furthermore, no association was found between HPV16, 18, and 33 and PCa [61]. Consistent results in a different study were produced, and once again found no association [44].

For these four studies, the conflicting results cannot always be explained. The Finnish and Nordic studies are very similar in terms of methodology and use the same threshold levels for the ELISA assays. The Nordic study includes three different cohorts from three different countries, Norway, Finland, and Sweden, and tests for associations between and within countries. This indicates that geographical region is also not a cause of differing results, as both used Finnish patients. The main difference between the two studies is the sample number, and the Nordic study likely had a higher statistical power that was able to exclude results because of chance. The inverse relationship between HPV18 seropositivity and PCa found in the Nordic study needs further analysis. The negative findings of the US studies could be down to the difference in timings between serum collection and diagnosis. In the 2010 study, blood was collected closer to diagnosis at the second visit. The 2007 study took blood between 1993 and 1995, and the follow-up period was a diagnosis in 2000. In contrast, the two Scandinavian studies collected serum several years before diagnosis and so an early HPV oncogenic mechanism may or may not have been detected.

Two studies that also meet the temporality criterion used archival formalin-fixed paraffin-embedded (FFPE) tissue samples rather than serum. The first study was conducted in an Australian population of men with benign prostate biopsies who, 1 to 10 years later, developed PCa [83]. In all, 52 sets of benign and PCa specimens from the same patients were collected, and PCR and IHC were used to analyse the samples. The study reported that there were no statistically significant differences in the prevalence of HPV L1 and E7 DNA (as assessed by PCR). However, differences in HPV E7 oncoprotein expression (as assessed by IHC) were highly significant, with much higher expression in the benign as compared to the later PCa in the same patient (*p* < 0.001). In contrast, a Swedish study conducted by Bergh et al. [62] used prostate tissue samples from 201 men with BPH who had undergone transurethral resection of the prostate (TURP) and subsequently developed PCa, along with 201 matched controls who did not develop PCa. A TURP is a type of surgical procedure that removes a portion of the prostate gland. Using PCR, no HPV DNA was detected in any of the samples. Dodd and colleagues also provided evidence that transcripts of the HPV E6/E7 viral genes may be detected in both benign and malignant tissues of the prostate [57].

The positive findings from studies that meet the temporality criteria are consistent with a ‘hit and run’ hypothesis for the role of HPV in PCa. This suggests that HPV acts early in the oncogenic process, supported by its presence in benign prostate tissue years before cancer diagnosis [83] and is reflected in positive serology results [81]. This hypothesis proposes that HPV infects cells transiently, and this begins the process of malignant transformation perhaps as a consequence of immune responses or HPV-induced DNA damage. However, the presence of HPV is not needed to maintain cancer and hence cells lose the viral genome, as shown by its lowered presence at later stages.

In conclusion, temporality is a key Bradford Hill criterion; however, it is hard to test for. As results between these studies are conflicting, further studies need to be conducted that meet this criterion to prove that exposure precedes disease.

### 4.6. Oncogenic Mechanism

To become cancerous, cells need to acquire specific characteristics, known as the “Hallmarks of Cancer” [109]. These include sustaining growth signals, evading natural growth blockers, avoiding cell death (apoptosis), becoming immortal, invading other tissues, inducing blood vessel growth (angiogenesis), changing cellular metabolism, and avoiding the immune system [109]. In addition to these established hallmarks, a recent review proposed additional emerging hallmarks and enabling characteristics, such as senescent cells, polymorphic microbiomes, non-mutational epigenetic reprogramming, and phenotypic plasticity [110]. Two factors that contribute to the development of these hallmarks are genome instability and inflammation [109]. HPV may play a role in the development of some of these hallmarks in prostate cells, suggested by its established role in causing cervical cancer and other malignancies. Understanding the hallmarks of human cancer and the factors that contribute to their development, such as genome instability and inflammation, is crucial in identifying potential mechanisms by which HPV may contribute to prostate oncogenesis, particularly through the action of the HPV oncoproteins. The most commonly proposed mechanism by which HPV could contribute to PCa involves the E6 and E7 oncoproteins. Many studies test the presence of the E6 and E7 regions of the HPV genome using specific PCR primers.

The HPV E6 and E7 oncoproteins play a crucial role in cancer development by inactivating host tumour-suppressor proteins, p53 and pRb, respectively, and subsequently inducing genome instability and leading to cell proliferation, immortalisation, and malignant transformation (reviewed by [111,112]). Another potential way in which the HPV E6 and E7 oncoproteins may contribute to the development of PCa is by creating a chronic inflammatory environment. Chronic inflammation has been identified as an enabling characteristic of cancer, and previous studies suggested that it plays a role in the development and metastasis of human cancers [113]. The E6/E7 proteins disrupt the interferon signalling pathway, which is the body’s first line of defence against viral infection. As a result, they suppress the action of the immune system, making it more difficult for the body to eliminate cancerous cells [29]. Additionally, chronic inflammation can damage DNA by creating oxygen-reactive species [82].

Many of the studies discussed in this review used benign prostatic hyperplasia (BPH) tissue as a control, but BPH is also associated with inflammation. This presents a limitation because using BPH patients as controls may make it difficult to draw meaningful conclusions if both conditions have a shared cause [60]. If inflammation is a common factor between the two conditions, it becomes challenging to distinguish the role of HPV in PCa. However, it is rare to find healthy prostates in the age group of interest, and BPH is the most common control used in studies. Consequently, there may be a significant difference in HPV DNA prevalence in healthy prostate tissue compared to cancer and BPH, but it is challenging to test for this.

In addition, to expand the scope of the investigation, microRNAs (miRNAs) and cell regulator proteins, such as survivin, Bcl-2, and c-Myc, can be tested. miRNAs are a family of small, endogenous non-coding RNAs that regulate a wide variety of biological processes and have been found to be dysregulated in a range of cancers [114]. They can act as either tumour suppressors or oncogenes. Tumour-secreted miRNAs (miR-21 and miR29a) have been shown to bind directly to human Toll-like receptor 8, activating a Toll-like receptor-mediated inflammatory response that can result in tumour growth and metastasis [115]. The study by Khatami et al. (2022) reported no significant association between the presence of HPV infection and PCa (*p* = 0.102) [31]. The study did observe different miRNA expressions in the HPV-positive PCa group compared to the normal prostate tissues of age-homogeneous healthy individuals (control): miRNA-21, -150-5p, and -155 levels were significantly upregulated. However, there was no statistically significant difference in the expression level of any selected cellular miRNAs between HPV-positive PCa samples and HPV-negative normal prostate tissues (*p* > 0.05). Therefore, based on this study alone, it cannot be concluded that HPV infection confers oncogenic potential associated with differing miRNA expression in PCa. The expression of miRNA-150-5p has been implicated in promoting cancer by encouraging cell migration and invasion; this miRNA is upregulated in recurrent ovarian cancer [116]. Another miRNA, miRNA-155, was also expressed in haematopoietic stem cells and several solid tumours, and it plays a crucial role in regulating the immune response [117,118,119]. In addition, miRNA-155 has been implicated in cervical cancer, where it promotes cell proliferation, migration, and invasion, ultimately allowing a cell to evade apoptosis [120]. Thus, the increased expression of these miRNAs in PCa with oncogenic potential may contribute to the development of hallmarks of cancer in HPV-positive PCa cells.

Specific cell regulator proteins were also found to be differentially expressed in the HPV-positive PCa group compared to the HPV-negative PCa group [31]. A statistically significant increase of approximately 1.8-fold to 4-fold in the mean levels of matrix metalloproteinase (MMP)-9, c-Myc, survivin, Bcl-2, and MMP-2 were observed. p53 was expressed at lower levels in the HPV-positive tissue samples compared to HPV-negative samples (fold-change: 0.22, *p*-value < 0.0001), which supports the role of HPV in the degradation of this crucial tumour suppressor in these tissues [31]. Survivin and Bcl-2 are both anti-apoptotic mediators and so promote cancer progression by allowing cells to survive, even when carrying mutations. c-Myc is an oncogene that is a ‘master regulator’ of cell growth and metabolism [121]. Overexpression of the c-Myc protein is present in over 70% of human cancers [122], highlighting its role in malignant transformation. The upregulation of c-Myc in HPV-positive tissue and significant association of c-Myc expression level with E7 and E6 expression levels in this study present the possibility of HPV indirectly affecting PCa development in this way. MMPs are the main enzymes involved in the extracellular matrix (ECM) breakdown. The ECM holds cells together and plays a role in cell growth and survival [123]. Cancer cells have to be able to degrade the ECM to invade nearby cells and metastasise, and increased MMP-2 and -9 levels in HPV-positive tissue may highlight an indirect role that HPV plays in this process.

According to Anwar et al. (1992), HPV18 infection was observed in 12 out of 15 HPV-positive PCa cases with bone metastasis, which accounts for 80% of the cases [52]. In addition, in a recent study by Fatemipour et al. (2021), it was suggested that HPV may play a role in promoting metastasis in PCa through various molecular mechanisms, as the expression levels of certain genes associated with metastasis, including N-cadherin, SLUG, and TWIST, were found to be higher in HPV-positive specimens, while the expression levels of other genes associated with tumour suppression, such as PTPN-13 and E-cadherin, were lower in HPV-positive specimens [124]. This finding supports the notion that HPV promotes metastasis. In cervical cancer, HPV18 infection has been linked to cervical small-cell neuroendocrine carcinomas, which exhibit aggressive behaviour, rapid metastasis, and high mortality rates [125]. However, it is not clear whether HPV18 infection is associated with metastasis to specific organs, and more research is needed to establish the role of HPV in this regard. On the one hand, Tu et al. (1994) found that HPV18 was only detected in one out of 17 pelvic lymph nodes in metastatic PCa (PCa) cases, which suggests that HPV may not play a role in metastasis [64]. Conversely, Ghasemian et al. (2013) reported that three out of five PCa patients infected with HPV developed distant metastasis, which suggests a possible role of HPV in promoting metastasis. These conflicting results underscore the need for further research in this specific area [29].

Several studies included in this review suggested that HPV may be linked to later stages of the disease, disputing the ‘hit and run hypothesis’ by associating virus presence with clinical data such as the Gleason score (GS). The GS is a system used to grade PCa, with higher grades indicating more abnormal cells and a greater likelihood of cancer growth and spread. Positive associations between HPV and high GS suggest that the virus may play a later role in carcinogenesis by increasing inflammation in prostate tissue, which may lead to tumour development and metastasis. However, the results are conflicting. The first study to support this hypothesis reported a significant increase in HPV infection levels with increasing GS (*p* < 0.005) [52]. This study used a small sample size, and the higher GS observed in PCa samples may be due to chance. More recent studies reporting a larger sample size have produced consistent results, with significant associations between HPV infection and high GS with *p* values of 0.014, 0.003, and 0.0008, respectively [27,35,73]. However, other studies have found no association between HPV infection and tumour aggressiveness represented by GS [39,43,48], and one study even found a low GS in its one HPV-positive sample out of sixty PCa samples investigated for HPV presence [49]. The studies that oppose the hypothesis that HPV may be involved in the later stages of PCa development and tumour maintenance, rather than being the primary cause of cancer initiation, have larger sample sizes but use heterogeneous study designs. Therefore, further research focusing on the clinical characteristics of the cancer is needed to confirm the role of HPV in later carcinogenic processes reflected by higher GS and to determine whether HPV is needed for tumour maintenance. Ideally, future studies should use the same HPV detection methodology. Several studies suggested that the prostate can act as a reservoir for HPVs with carcinogenic potential, but the presence of HPV does not necessarily imply a direct causal relationship with PCa.

Recent investigations have focused on the emergence of apolipoprotein B mRNA editing enzyme, catalytic-polypeptide-like (APOBEC) enzymes as a protective mechanism against viral infections [126,127,128]. Studies have suggested that the oncogenic effect of HPV may not be a direct result of viral infection, but rather an indirect consequence of the ability of HPV to inhibit the protective function of APOBEC enzymes [126,127]. APOBEC mutations have been linked to certain human cancer genomes, and the APOBEC3A/B deletion polymorphism has been associated with a higher risk of some cancers, including PCa [128,129,130,131]. Specifically, changes in the protective effects of APOBEC3B against oncogenic viruses can occur, leading to host genome instability and cancer progression after HPV viral DNA integration [126,127,132]. The intricate relationship between viruses and APOBEC in cancer development is further complicated by the presence of two viruses, HPV and Epstein–Barr virus (EBV), which have both been identified in PCa [78]. The negative impact of EBV on the integrity of APOBEC adds an additional layer to this complexity [132]. Therefore, it appears that the main mechanism by which HPV influences PCa is indirect, involving the inhibition of enzymes such as APOBEC3B that would otherwise help protect against the harmful effects of viruses.

Despite these findings, the role of HPV in prostate carcinogenesis remains unclear, as there are conflicting reports among studies. The ‘hit and run’ hypothesis opposes the later-acting mechanism, which is observed by correlation with a higher GS and metastasis. The use of immunohistochemistry (IHC) proposes interesting mechanisms for how HPV may interact with cellular components, but more research is needed to confirm these findings [31]. Moreover, HPV infection may contribute to inflammation in the gland, but the use of BPH as a control makes it challenging to distinguish this role because of inflammation possibly being part of the aetiology of both conditions.

## 5. Consistency

Various studies examining this topic have yielded inconsistent results, which may be due in part to differences in methodology. While there have been previous discussions about different techniques for detecting HPV, this section will specifically examine discrepancies in the collection and storage of tissue samples (Table 4).

### 5.1. Consistency in Tissue Collection

The initial investigation on HPV DNA in prostate tissue relied primarily on TURP for sample collection, with suprapubic prostatectomy (SPP) used for two samples [47]. The study found high HPV detection rates: 4 out of 4 (100%) cases of PCa, and 14 out of 15 (93%) BPH controls tested positive. In a subsequent larger study by the same researchers, HPV detection rates were still relatively high, but lower than the first study: 56% of PCa cases, 61% of BPH controls, and 20% of healthy tissue controls tested positive [50]. TURP and SPP were again the primary collection methods, but there were concerns that TURP might introduce HPV from the male urethra into the prostate tissue, leading to an overestimation of HPV presence. The male urethra is susceptible to HPV infection, and HPV DNA may be present in this area [133]. This issue prompted other studies to explore other tissue collection methods to minimise the potential for contamination.

Another study used a microdissection approach that excluded urethral mucosa and found no HPV DNA in PCa cases [67], while another found HPV DNA in BPH specimens obtained via TURP and SPP, suggesting that prostate tissue may be the source of HPV. However, other studies that used TURP did not find any HPV DNA [51,59,62,66,82], indicating that contamination may be a significant confounding factor.

It is conceivable that high occurrence in the initial detection of HPV and PCa was due to contamination from urethral tissue. Nonetheless, it is more likely that the small sample size was the actual cause, as evidenced by other TURP-based studies that did not produce positive results. Most studies in this review shifted away from the TURP method, particularly for PCa cases, which implies that contamination is not a significant confounding variable to consider in this review.

#### Consistency in Tissue Storage

Another difference in study methodology is the way in which tissue is stored. Most studies used FFPE blocks, fresh, or frozen tissue. In the earlier studies, fresh or frozen tissue was primarily used, but most studies in this review used FFPE blocks as the storage method (Table 4). The use of FFPE samples allows superior morphological assessment of tissue for hybridisation analysis and less chance of carry-over contamination during PCR [55]. However, the fixation process can influence the yield of high-quality DNA, which may affect its suitability for PCR [134]. This is probably not an issue in this review as most studies tested the quality of DNA extracted from samples using a reference human gene, such as beta-globin. Furthermore, when testing for this, no significant difference was seen in HPV prevalence between FFPE and fresh frozen samples (*p* = 1.00) [55].

In conclusion, the studies reviewed here are inconsistent with each other in terms of HPV detection rates, HPV detection methodology, and tissue collection and storage methodology. Although some studies found these differences to have non-significant effects on the results, it is not clear what the true reasons are for discrepancies among studies. Perhaps a review that focuses on studies using solely the same techniques would mean the consistency criterion could be met, but this would require many further studies.

## 6. Biological Gradient

The original Bradford Hill criteria state that a dose–response relationship supports a causal association. However, for HPV and PCa, demonstrating different exposure dosages is difficult as most studies only report whether the virus is present. A few studies in this review did consider the HPV copy number per cell [32,33,50,56], but the results were inconsistent, likely because of the heterogeneity of prostate specimens [47]. The L1 consensus primers GP5+/6+ can detect 1 HPV DNA copy per 100 PCa cells, meaning it is sensitive enough to detect low copy numbers of HPV DNA [59]. One study that used ISH stated that the probe used could detect 10 copies, and hence a weak positive ISH analysis may be due to the assay not being sensitive enough to detect low copy numbers [25]. Therefore, a dose–response relationship cannot be concluded. It is not a crucial causal criterion since the ‘hit and run’ hypothesis suggests that a high viral load may be unnecessary for prostate carcinogenesis.

## 7. Experimental Evidence

Bradford Hill described this criterion as evidence drawn from experimental manipulation [98]. In this case, manipulation refers to removal of the exposure and observing if the risk declines. Cessation of exposure, such as treating HPV to see if PCa rates decrease, was also not conducted in this review. This is an example of an intervention study, whereas the ones in this review are observational: case-control and cohort studies. However, it is possible to link this criterion to vaccination programmes against HPV and cessation of exposure in this regard. Information on rates of PCa following an HPV vaccination programme are not available. However, this is an effective measure of prevention against cervical cancer [135,136]. Hence, rates of PCa in countries following HPV vaccination could be a way forward in the future to provide experimental evidence on HPV as a cause of PCa.

To conclude, in this review, experimental manipulation of HPV as an exposure was not tested for and cannot be commented on for causal inference in PCa. Notably, expression of HPV16 E6 and E7 was shown to immortalise prostate epithelial cells [137], which provides strong experimental evidence that HPV oncoproteins allow prostate cells to avoid normal cell control mechanisms.

## 8. Conclusions

Although the evidence available to date provides interesting insights into the potential role of high-risk HPV in PCa, it is not yet conclusive enough to meet key Bradford Hill criteria such as strength of association, consistency, and coherence. Additionally, the complex nature of HPV transmission and oncogenic mechanisms, coupled with the difficulty of testing criteria such as temporality, biological gradient, and experiment in this context, make it challenging to draw definitive conclusions.

While there are some similarities between PCa and cervical cancer, they also have significant differences that limit the extent to which analogies can be drawn between the two. Although the identification and transmission of HPV provide valuable information regarding the virus’s plausibility as a causative agent in PCa, the conflicting nature of the literature prevents us from drawing a definitive conclusion about the causal role of HPV in this disease.

However, it is possible that the prostate serves as a reservoir for HPV transmission. If high-risk HPV does play a role in prostate carcinogenesis, it is likely through the actions of the viral oncoproteins, the promotion of inflammation, which could potentially contribute to the oncogenic process at various stages, and/or damage to host DNA. Further studies are required to gain a better understanding of this topic, potentially focusing on homogeneous study designs and result analyses. Therefore, while the evidence to date suggests a possible causal role for HPV in PCa, it is not yet strong enough to use the term “highly likely” without further research to support this claim.

## Figures and Tables

**Table 1:** The inclusion and exclusion criteria used during abstract screening.

Inclusion Criteria	Exclusion Criteria
Primary studies	Secondary data (e.g., Meta-Analysis and Systematic reviews)
Tissues only infected with HPV	Coinfections
Male participants over the age of 18	Male participants aged <18
Paper in English	Studies published languages other than English
	Duplicated papers

**Table 2:** Assessment of case-control study quality using the Newcastle–Ottawa Quality Assessment Scale. 1—Is the case definition accurate? 2—Representativeness of the cases. 3—Selection of controls. 4—Definition of controls. 5—Comparability of cases and controls of the basis of the design or analysis (*—star awarded when study tested the quality of its samples). 6—Ascertainment of exposure. 7—Same method of ascertainment for cases and controls. S/N—Study number.

S/N	Study	Selection				Comparability	Exposure		Total
		1′	2	3	4	5	6	7	
1	Medel Flores et al. (2018) [24]	*	*	-	*	*	*	*	6
2	Chen et al. (2011) [25]	*	*	-	*	*	*	*	6
3	Aghakhani et al. (2011) [26]	*	*	-	*	*	*	*	6
4	Zhao et al. (2017) [27]	*	*	-	*	-	*	*	5
5	Tachezy et al. (2012) [28]	*	*	-	*	*	*	*	6
6	Ghasemian et al. (2013) [29]	*	*	-	*	*	*	*	6
7	Rodriguez et al. (2016) [30]	*	*	-	*	*	*	*	6
8	Khatami et al. (2022) [31]	*	*	-	*	*	*	*	6
9	Rotola et al. (1992) [32]	*	*	-	*	*	*	*	6
10	Moyret-Lalle et al. (1995) [33]	*	*	-	*	*	*	*	6
11	Atashafrooz et al. (2016) [34]	*	*	-	*	*	*	*	6
12	Singh et al. (2015) [35]	*	*	-	*	*	*	*	6
13	Sarkar et al. (1993) [36]	*	*	-	-	*	*	*	5
14	Noda et al. (1998) [37]	*	*	-	*	*	*	*	6
15	Korodi et al. (2005) [38]	*	*	*	*	-	*	*	6
16	Carozzi et al. (2004) [39]	*	*	-	*	*	*	*	6
17	Adami et al. (2003) [40]	*	*	*	*	-	*	*	6
18	Leiros et al. (2005) [41]	*	*	-	*	*	*	*	6
19	Wideroff et al. (1996) [42]	*	*	-	*	*	*	*	6
20	Martinez-Fierro et al. (2010) [43]	*	*	*	*	*	*	*	7
21	Sutcliffe et al. (2010) [44]	*	*	*	*	-	*	*	6
22	Silvestre et al. (2009) [45]	*	*	-	*	*	*	*	6
23	Michopoulou et al. (2014) [46]	*	*	-	*	*	*	*	6
24	McNicol and Dodd (1990) [47]	*	*	-	*	*	*	*	6
25	Rosenblatt et al. (2003) [48]	*	*	*	*	-	*	*	6
26	Aydin et al. (2017) [49]	*	*	-	*	*	*	*	6
27	McNicol and Dodd (1991) [50]	*	*	-	*	*	*	*	6
28	Masood et al. (1991) [51]	*	*	-	*	-	*	*	5
29	Anwar et al. (1992) [52]	*	*	-	*	-	*	*	5
30	Terris and Peehl (1997) [53]	*	*	-	-	*	*	*	5
31	Suzuki et al. (1996) [54]	*	*	-	*	-	*	*	5
32	Ibrahim et al. (1992) [55]	*	*	-	*	*	*	*	6
33	Serth et al. (1999) [56]	*	*	-	*	*	*	*	6
34	Dodd et al. (1993) [57]	*	*	-	*	*	*	*	6
35	Salehi and Hadavi (2012) [58]	*	*	-	*	*	*	*	6
36	Strickler et al. (1998) [59]	*	*	-	*	*	*	*	6
37	Hrbacek et al. (2011) [60]	*	*	-	*	-	*	*	5
38	Sutcliffe et al. (2007) [61]	*	*	*	*	-	*	*	6
39	Bergh et al. (2006) [62]	*	*	-	*	*	*	*	6
40	Groom et al. (2012) [63]	*	*	-	*	*	*	*	6
41	Tu et al. (1994) [64]	*	*	-	*	*	*	*	6
42	Sitas et al. (2007) [65]	*	*	-	*	-	*	*	6
43	Anderson et al. (1997) [66]	*	*	-	*	*	*	*	6
44	Effert et al. (1992) [67]	*	*	-	-	-	*		3
45	Afshar et al. (2013) [68]	*	*	*	-	-	*	*	5
46	Araujo-Neto et al. (2016) [69]	*	*	-	-	-	*	*	4
47	Balis et al. (2007) [70]	*	*	-	-	-	*		3
48	Mokhtari et al. (2013) [71]	-	*	-	*	*	-	*	4
49	Pascale et al. (2013) [72]	*	*	-	-	-	*	-	3
50	Abumsimir et al. (2022) [73]	*	*	-	-	-	*	-	3
50	Nahand et al. (2020) [74]	*	*		-	*	*	*	5
51	Pereira et al. (2023) [75]	*	*	-	-	-	*	-	3
52	Rodriguez et al. (2015) [76]	*	*	-	-	-	*	-	3
53	Yow et al. (2014) [77]	*	*	-	-	-	*		3
54	Whitaker et al. (2012) [78]	*	*	-	*	-	*	*	5
55	Ahmed et al. (2023) [79]	*	*	*	*	*	*	*	7
56	Chang et al. (2023) [80]	*	*	*	*	*	*	*	7

**Table 3:** Assessment of cohort study quality using the Newcastle–Ottawa Quality Assessment Scale. 1—Representativeness of the exposed cohort. 2—Selection of the non-exposed cohort. 3—Ascertainment of exposure. 4—Demonstration that outcome of interest was not present at start of study. 5—Comparability of cohorts based on the design or analysis (*—star awarded when study tested the quality of its samples). 6—Assessment of outcome. 7—Was followed-up long enough for outcomes to occur (3 years)? 8—Adequacy of follow-up of cohorts.

S/N	Study	Selection				Comparability	Outcome			Total Score
		1	2	3	4	5	6	7	8	
1	Dillner et al. (1998) [81]	*	*	*	-	-	*	*	-	5
2	Gazzaz and Mosli (2009) [82]	*	*	*	*	-	*	-	-	5
3	Glenn et al. (2017) [83]	*	-	*		-	-	*	*	4
4	Dennis et al. (2009) [84]	*	*	*	*	*	*	-	-	6

**Table 4:** PCR studies included in this review. Formalin-fixed paraffin-embedded, FFPE; paraffin-embedded, PE; radical prostatectomy, RP; simple prostatectomy, SP; total prostatectomy, TP; suprapubic prostatectomy, SPP; subcapsular prostatectomy, SCP; transurethral resection of the prostate, TURP; radical retropubic prostatectomy, RRP; open prostatectomy, OP; transrectal biopsy, TRB; transperineal biopsy, TPB; transvesical prostatectomy, TVP; Transrectal ultrasound, TRUS.

				Subjects			HPV Types			
S/N	Author	HPV Detection	Collection (Storage)	No.	Type	HPV	6	16	18	Other
1	Medel Flores et al. (2018) [24]	L1 PCR consensus primers E6/E7 PCR for HPV 16HPV16, 18, 31, 33, 52b, 58	RP (FFPE)	189	PCa cases	37 (20%)	6	7	8	52 (17), 58 (12)
			RP (FFPE)	167	BPH controls	16 (10%)		1	3	52 (16), 58 (5)
2	Pascale et al. (2013) [75]	E7 IHC staining L1 PCR consensus primer	Surgery, not fine needle biopsies (FFPE)	150	PCa cases	19 (13%)		9		
3	Chen et al. (2011) [25]	L1 PCR consensus primer Type-specific PCR primer for HPV 18 ISH for HPV 18	Collection not specified (snap frozen)	51	PCa cases	7 (14%)			7	
			Collection not specified (snap frozen)	11	BPH controls	3 (27%)			3	
4	Aghakhani et al. (2011) [26]	L1 PCR consensus primers	RP, TURP (FFPE)	104	PCa cases	13 (13%)	1	7	3	11 (2)
			RP, TURP (FFPE)	104	BPH controls	8 (8%)	1	3	2	11 (2)
5	Tachezy et al. (2012) [28]	L1 PCR consensus primers	RRP (FFPE)	51	PCa cases	1 (2%)				42 (1)
			11 SP, 84 TURP (FFPE)	95	BPH controls	2 (2%)		1		1 unknown
6	Mokhtari et al. (2013) [71]	IHC staining	Collection not specified (PE)	30	PCa cases	3 (10%)				
			Collection not specified (PE)	90	BPH controls	1 (1%)				
7	Balis et al. (2007) [70]	L1 PCR consensus primer Type-specific PCR primers for HPV 11, 16, 18, 33	Collection not specified (22 FFPE, 20 fresh frozen)	42	PCa cases	2 (5%)				Unknown
8	Ghasemian et al. (2013) [29]	L1 PCR consensus primers	Collection not specified (FFPE)	29	PCa cases	5 (17%)				
			Collection not specified (FFPE)	167	BPH controls	8 (5%)				
9	Rodriguez et al. (2016) [30]	INNO-LiPA HPV kit—L1 and 28 HPV genotypes	OP (FFPE)	62	PCa cases	12 (20%)				11 (46.1%), 51, 52, and 66 (15.4%)
			TURP (FFPE)	25	BPH controls	1 (4%)			1	
10	Khatami et al. (2022) [31]	L1 PCR consensus primer	Collection not specified (snap frozen)	73	PCa cases	21 (29%)	1	9	7	11 (1), 33 (3)
			Collection not specified (snap frozen)	39	Healthy controls	7 (8%)		3	3	11 (1)
11	Rotola et al. (1992) [32]	E6 PCR for HPV 6/11, 16	Collection not specified (snap frozen)	8	PCa cases	N/A	4	6		11 (4)
			Collection not specified (snap frozen)	17	BPH controls	N/A	11	14		11 (11)
12	Moyret-Lalle et al. (1995) [33]	E6 PCR for HPV 16HPV16 and 18	Collection not specified (snap frozen)	17	PCa cases	9 (53%)		9		
			Collection not specified (snap frozen)	22	BPH controls	7 (32%)		7		
13	Atashafrooz et al. (2016) [34]	Real-Time PCR HPV detection/genotyping assay kit—13 genotypes	Collection not specified (PE)	100	PCa cases	20 (20%)	1			16/18 (8), 31/33 (6), 54 (2), 6/11 (3)
			Collection not specified (PE)	100	BPH Controls	8 (8%)	2			16/18 (1), 31/33 (1), 6/11 (4)
14	Araujo-Neto et al. (2016) [69]	L1 PCR consensus primers E6/E6 PCR for HPV 16HPV16	RP (fresh frozen)	104	PCa cases	0 (0%)				
15	Singh et al. (2015) [35]	L1 PCR consensus primers Type-specific PCR primers for HPV 6, 11, 16, 18	Collection not specified (storage not specified)	95	PCa cases	39 (41%)	2	30	6	11 (1)
			Collection not specified (storage not specified)	55	BPH controls	11 (20%)	6	3	1	11 (1)
16	Sarkar et al. (1993) [36]	E6/E7 PCR for HPV 6, 11, 16, and 18 Southern blot hybridisation	Surgical resection, not TURP (PE)	23	PCa cases	3 (13%)		3		
			Surgical resection, not TURP (PE)	23	PIN controls	0 (0%)				
17	Noda et al. (1998) [37]	PCR primers for LCR and E7 for HPV 16HPV16, 18, 31, 33, 52, 58	TP (FFPE)	38	PCa cases	0 (0%)				
			10 SCP, 61 TURP (FFPE)	71	BPH controls	3 (4%)		3		
18	Carozzi et al. (2004) [39]	L1 PCR consensus primer E6/E7 PCR for HPV types 16, 18, 31, 33, 35, 45, 52, 58 Hybridisation	TPB (formalin)	26	PCa cases	17 (65%)	1	3	3	58 (4)
			TPB (formalin)	25	BPH controls	12 (48%)	1		2	53 (4)
19	Leiros et al. (2005) [41]	Type-specific PCR primers for HPV 6, 11, 16, 18 L1 PCR consensus primer Southern blot hybridisation	TRB (FFPE)	41	PCa cases	17 (42%)		5		11 (2)
			TRB (FFPE)	30	BPH controls	0 (0%)				
20	Wideroff et al. (1996) [42]	L1 PCR consensus primers E6 PCR for HPV 6, 11, 16, 18, 31, 33, 45 Hybridisation	TURP, RP, excision biopsy (FFPE)	56	PCa cases	7 (13%)				
			TURP (FFPE)	42	BPH controls	4 (10%)				
21	Martinez-Fierro et al. (2010) [43]	L1 PCR consensus primers Linear Array HPV Genotyping Test	TRB, TURP (storage not specified)	55	PCa cases	11 (20%)				
			TRB, TURP (storage not specified)	75	Non-PCa controls	4 (5%)				
22	Silvestre et al. (2009) [45]	L1 PCR consensus primer Linear Array HPV Genotyping Test	Collection and storage not specified	65	PCa cases	2 (3%)		2		84 (coinfection)
			Collection and storage not specified	6	BPH controls	0 (0%)				
23	Michopoulo et al. (2014) [46]	L1 PCR consensus primer	Collection not specified (FFPE)	50	PCa cases	8 (16%)		2	4	31 (1), unknown (1)
			Collection not specified (FFPE)	30	Healthy controls	1 (3%)				Unknown (1)
24	McNiol and Dodd (1990) [47]	E6 PCR for HPV 16HPV16 and 18	2 SPP, 17 TURP (fresh frozen)	4	PCa cases	4 (100%)		4		
				15	BPH controls	14 (93%)		11		16 + 18 (3)
			Autopsy (fresh frozen)	5	Healthy controls	1 (20%)		1		
25	Aydin et al. (2017) [49]	L1 PCR consensus primer HPV sign^®^ Q24 for genotyping	RP (FFPE)	60	PCa cases	1 (2%)				57
			TVP (FFPE)	36	BPH controls	0 (0%)				
26	McNiol and Dodd (1991) [50]	E6 PCR for HPV 16HPV16 and 18 Hybridisation	TURP, SPP (fresh frozen)	27	PCa cases	14 (52%)		14	1	
			TURP, SPP (fresh frozen)	56	BPH controls	34 (63%)		34	3	
			Autopsy (fresh frozen)	5	Healthy controls	1 (20%)		1		
27	Masood et al. (1991) [51]	In situ hybridisation for HPV 6, 11, 16, 18, 31, 33, 35	Core needle biopsy, TURP (FFPE)	20	PCa cases	0 (0%)				
			Core needle biopsy, TURP (FFPE)	20	BPH controls	0 (0%)				
28	Anwar et al. (1992) [52]	E6 PCR for HPV 16HPV16, 18, 33	TURP, SPP (FFPE)	68	PCa cases	28 (41%)		11	7	33 (5)
			TURP, SPP (FFPE)	10	BPH controls	0 (0%)				
			Autopsy (FFPE)	10	Healthy controls	0 (0%)				
29	Rodriguez et al. (2015) [76]	L1 PCR consensus primer Type-specific PCR for 19 HPV genotypes	Collection not specified (FFPE)	69	PCa cases	0 (0%)				
30	Terris and Peehl (1997) [53]	L1 PCR consensus primer E6 PCR for HPV 16HPV16	RRP (FFPE)	53	PCa cases	12 (23%)		12		
				41	Peripheral benign tissue	15 (37%)		15		
				37	Healthy controls	6 (16%)		6		
31	Suzuki et al. (1996) [54]	L1 PCR consensus primer	29 TP, 22 autopsy (storage not specified)	51	PCa cases	8 (16%)		8		
32	Ibrahim et al. (1992) [55]	L1 PCR consensus primer ISH	RP, TURP, TRB (FFPE and fresh frozen)	40	PCa cases	6 (15%)		6		
			RP, TURP, TRB (FFPE)	12	BPH controls	0 (0%)				
			RP, TURP, TRB (FFPE)	17	Healthy controls	2 (12%)		2		
33	Serth et al. (1999) [56]	E6 PCR for HPV 16HPV16	RP (snap frozen)	47	PCa cases	10 (21%)		10		
			TVP (snap frozen)	37	BPH controls	1 (3%)		1		
34	Dodd et al. (1993) [57]	Reverse transcription PCR for E6/E7 mRNA of HPV16	Collection not specified (fresh frozen)	7	PCa cases	3 (43%)		3		
			Collection not specified (fresh frozen)	10	BPH controls	5 (50%)		5		
35	Salehi and Hadavi (2012) [58]	L1 PCR consensus primer	Collection not specified (snap frozen)	68	PCa cases	3 (4%)				
			Collection not specified (snap frozen)	85	BPH controls	0 (0%)				
36	Abumsimir et al. (2022) [73]	L1 PCR consensus primers	Biopsies (fresh)	50	PCa cases	8 (16%)			8	
37	Strickler et al. (1998) [59]	L1 PCR consensus primers E6 PCR for HPV 11, 16, 18, 51, 61 Southern blot hybridisation	Surgery, TURP (fresh frozen)	63	PCa cases	0 (0%)				
			Surgery, TURP (fresh frozen)	61	BPH controls	0 (0%)				
38	Glenn et al. (2017) [83]	L1 PCR consensus primer E7 PCR for HPV 16HPV16 and 18 IHC for E7 oncoprotein	Collection not specified (FFPE)	28	PCa cases	L1 8 (29%) E7 19 (68%) E7P 8 (29%)				
				28	BPH controls—years before	L1 13 (46%) E7 23 (82%) E7P 23 (82%)				
39	Bergh et al. (2006) [62]	L1 PCR consensus primer	TURP (FFPE)	201	PCa cases	0 (0%)				
			TURP (FFPE)	201	BPH controls	0 (0%)				
40	Groom et al. (2012) [63]	INNO-LiPA HPV Genotyping kit Hybridisation to HPV 16HPV16	Collection and storage not specified	100	PCa cases	1 (1%)		1		
			Collection and storage not specified	62	Healthy controls	0 (0%)				
41	Tu et al. (1994) [64]	L1 PCR consensus primer Hybridisation	RP (FFPE)	43	PCa cases	1 (2%)		1		
			Collection not specified (snap frozen)	17	Metastatic pelvic lymph nodes	1 (6%)			1	
			RRP (not specified)	1	Normal prostate	0 (0%)				
42	Effert et al. (1992) [67]	E6 PCR for HPV 16HPV16 and 18	RP (FFPE)	30	PCa cases	0 (0%)				
43	Gazzaz and Mosli (2009) [82]	Hybridisation using Hybrid Capture 2 kit	TURP, TRB (fresh)	6	PCa cases	0 (0%)				
			TURP, TRB (fresh)	50	21 BPH, 29 nodular hyperplasia	0 (0%)				
44	Anderson et al. (1997) [66]	L1 PCR consensus primer E6 and E2 ORF PCR for HPV 16HPV16	TURP (fresh frozen)	14	PCa cases	0 (0%)				
			TURP (fresh frozen)	10	Benign controls	0 (0%)				
45	Pereira et al. (2023) [75]	L1 PCR consensus primer for HPV 16HPV16, 18, 56, 59 and 66.	TRUS Biopsies	162	PCa cases,	10 (6.2%)				
46	Afshar et al. (2013) [68]	PCR and INNo- Lipa assays	Paraffin embedded blocks	410	PCa cases	108 (26.34%)	53	11		11 (10), others (34)
47	Nahand et al. (2020) [74]	L1 and E7 PCR consensus primer and INNO-LiPA HPV Genotyping Kit	Surgery	58	PCa cases	19 (32.7%)		9	6	33 (3)
48	Yow et al. (2014) [77]	PapType High-Risk (HR) HPV Detection and Genotyping kit	TRUP (FFPE) RP (FFPE)	221	PCa cases	0 (0%)				
49	Whitaker et al. (2012) [78]	L1 PCR consensus primer, and In Situ PCR	Collection not specified (FFPE; Fresh frozen)	10	PCa cases	7 (70%)			1	
50	Ahmed et al. (2023) [79]	HPV-HCR Genotype-Eph kit	Biopsies (Fresh)	49	PCa cases, Benign controls	16 (32.7%)		4		33, 35, 45, 52, 56, 58
51	Chang et al. (2023) [80]	Cobas 4800 HPV Test and DR HPV Genotyping IVD Kit	FFPE	178	PCa cases, Benign controls	12 (6.7%)			2	52 (1), 53 (3), 62 (1), others (5)

**Table 5:** Serology studies included in this review.

S/N	Author	Year	Pathogen Studied	PCa Present	% Seropositive	No. of Controls without PCa	% Seropositive	RR/OR	95% CI	Evidence of Association?	Method
1	Chen et al. [25]	2011	HPV 16HPV16, 18, 31, 33, 52, 58	26/53	49	35/104	33.7	OR = 0.71–4.16	0.18–48.0	No	Fluorescent assay
2	Zhao et al. [27]	2017	HPV 16HPV16	48/75	64	14/80	17.5	N/A	N/A	Yes (*p* < 0.001)	Peptide microarray
3	Tachezy et al. [28]	2012	HPV 16HPV16, 18, 31, 33	14/51	27	172	21.5	1.44	0.69–2.97	*p* = 0.329	ELISA
4	Dillner et al. [81]	1998	HPV 16HPV16, 18, 33	31/165	18.8	35/290	12.1	RR = 2.59, 2.38, 0.66	1.17–5.75 0.75–7.58 0.26–1.66	Yes for HPV 16HPV16 and 18 (*p* < 0.001)	ELISA
5	Korodi et al. [38]	2005	HPV 16HPV16, 18, 33	107/799	13.4	363/2596	14.0	OR 0.94	0.74–1.19	No	ELISA
6	Adami et al. [40]	2003	HPV 16HPV16, 18, 33	129/238	54	105/210	50	OR = 0.7, 0.9, 1.6	0.4–1.3, 0.5–1.9, 1.0–2.7	Yes for HPV 33	ELISA
7	Sutcliffe et al. [44]	2010	HPV 16HPV16, 18, 33	180/616	29.2	179/616	29	OR 1.07, 0.87, 1.15	0.77–1.48 0.47–1.63	No	ELISA
8	Rosenblatt et al. [48]	2003	HPV 16HPV16, 18	81/642	12.6	64/570	11.3	OR = 1.06, 1.36	0.71–1.57 0.69–2.69	No	ELISA
9	Strickler et al. [59]	1998	HPV 16HPV16	1/63	1.6	7/144	4.9	N/A	N/A	No (*p* = 0.44)	ELISA
10	Hrbacek et al. [60]	2011	HPV 16HPV16, 18, 31, 33	50/329	15.2	33/105	31	OR = 0.48, 023, 073, 0.43	0.21–1.13 0.09–0.61 0.32–1.83 0.13–1.48	No	ELISA
11	Sutcliffe et al. [61]	2007	HPV 16HPV16	144/691	20.8	145/691	21	OR = 0.83, 1.04, 1.14	0.57–1.23 0.66–1.64 0.76–1.72	No	ELISA
12	Sitas et al. [65]	2007	HPV 16HPV16	59/205	28.78	173/673	25.71	OR 1.33	0.86–2.07	No	ELISA
13	Dennis et al. [84]	2009	HPV 16HPV16 and 18	50/267	19	45/267	17	OR 1.33	0.73–1.75	No	ELISA

**Table 6:** Included PCR studies with no significant differences in the presence of HPV and the risk of developing PCa.

S/N	Author	OR (95% Cl)	*p*-Value
1	Chen et al. (2011) [25]		*p* > 0.05
2	Aghakhani et al. (2011) [26]	-	*p* > 0.05
3	Khatami et al. (2022) [31]	2.01 (0.8–5.68)	*p* = 0.102
4	Atashafrooz et al. (2016) [34]	-	*p* = 0.413
5	Noda et al. (1998) [37]	-	*p* = 0.19
6	Wideroff et al. (1996) [42]	1.36 (0.37, 4.98)	*p* > 0.05
7	Michopoulou et al. (2014) [46]	5.52 (0.66–45.6)	*p* = 0.086
8	Rotola et al. (1992) [32]	-	*p* > 0.05
9	McNicol and Dodd (1991) [50]		*p* > 0.05
10	Ibrahim et al. (1992) [55]	-	*p* = 0.343
11	Salehi and Hadavi (2012) [58]		*p* = 0.71
12	Nahand et al. (2020) [74]		*p* = 0.078
13	Periera et al. (2023) [75]		*p* = 0.487
14	Ahmed et al. (2023) [79]		*p* > 0.05
15	Chang et al. (2023) [80]		*p* > 0.005
